# Application of a multidimensional and process-oriented evaluation system in PELD bridging therapy for lumbar spinal infections: a self-controlled retrospective study of 27 cases

**DOI:** 10.3389/fcimb.2026.1920706

**Published:** 2026-08-04

**Authors:** Yanan Wang, Xingsheng Yu, Jianhua Li, Yang Yang, Zhengqi Chang

**Affiliations:** Department of Orthopedics, The 960th Hospital of PLA, Jinan, China

**Keywords:** lumbar spine infection, modified POLA classification, multidimensional evaluation system, nutritional risk, percutaneous endoscopic lumbar discectomy, retrospective study

## Abstract

**Background:**

Conventional cure rates are too stringent for percutaneous endoscopic lumbar discectomy (PELD)-based endoscopic debridement and drainage as a minimally invasive bridging procedure for spinal infections; a multidimensional evaluation system is needed.

**Methods:**

This single-center retrospective self-controlled study included 27 patients with modified Pola classification (POLA) type IV lumbar infections who underwent Stage I PELD-based endoscopic debridement and drainage followed by Stage II definitive surgery. The procedure was evaluated as a planned bridging intervention within an integrated treatment pathway rather than as an isolated curative treatment. A multidimensional system assessed biochemical, clinical (pain, appetite, temperature), imaging (magnetic resonance imaging (MRI)-based modified POLA), and anesthesiologist scores. Paired t-tests and Wilcoxon tests were used for within-patient comparisons.

**Results:**

Stage I PELD-based endoscopic debridement and drainage was completed in all patients (mean operation time, 82.4 min; mean blood loss, 18.5 mL; culture positivity, 55.6%). At final follow-up, visual analog scale (VAS) back-pain score decreased from 8.15 to 0.67, Oswestry Disability Index (ODI) decreased from 76.15% to 11.78%, and 36-Item Short Form Health Survey (SF-36) increased from 26.26 to 83.74 (all P<0.001). C-reactive protein (CRP) and erythrocyte sedimentation rate (ESR) decreased before Stage II surgery, body temperature improved, appetite improved in 63%, and modified POLA grading improved. These findings suggested clinical stabilization during the bridging window, whereas nutritional indicators (albumin, hemoglobin, and body mass index (BMI)) did not significantly change.

**Conclusion:**

PELD-based endoscopic debridement and drainage appears to be a feasible minimally invasive bridging strategy for modified POLA type IV lumbar infections. The observed improvements should be interpreted as preliminary evidence of clinical stabilization during staged treatment rather than proof of an independent curative effect of PELD. The proposed multidimensional evaluation system provides an exploratory framework for assessing early bridging response and requires further validation in larger controlled cohorts.

## 1 Introduction

Spinal infection, including pyogenic spondylitis and spinal tuberculosis, is the most common type of spinal infection, accounting for about 50% of cases (Vcelak et al., 2014). It poses a challenge in the field of spinal surgery due to increasing incidence related to life expectancy, risk factors, and complications (Zarghooni et al., 2012; Kramer et al., 2023; Bal et al., 2025). The pathological process involves microbial invasion of the vertebral body, intervertebral disc, and surrounding tissues, leading to local destruction, abscess formation, severe lower back pain, neurological damage (Placide and Reznicek, 2025), and even systemic sepsis, significantly threatening patients’ quality of life and health. Patients often present with varying degrees of malnutrition or nutritional risk due to chronic consumption issues, infectious fever, and pain (Bohl et al., 2016), which can weaken the immune system, delay tissue repair, increase complication rates, and mortality, creating a vicious cycle (Gerstmeyer et al., 2025).

While open surgery has traditionally been used for patients with poor response to conservative treatment (Rezai et al., 1999; Pola et al., 2012), significant abscesses, spinal instability, or nerve compression, it can cause substantial trauma, bleeding, and disrupt spinal stability, serving as a major physiological stressor that may exacerbate nutritional depletion and metabolic disturbances, delaying postoperative recovery (Guerado and Cerván, 2012).

With the rapid development of minimally invasive spinal surgery techniques (Patel et al., 2020), percutaneous endoscopic lumbar discectomy (PELD) has been widely applied in the treatment of degenerative diseases like lumbar disc herniation (Gibson et al., 2012; Gunjotikar et al., 2024), offering advantages of minimal trauma, reduced bleeding, and faster recovery (Yang et al., 2014). In recent years, this technique has been explored for the treatment of spinal infections (Rajkumar and Li, 2024; Moschos et al., 2025), showing theoretical advantages in precise lesion debridement, abscess drainage, and obtaining tissue for microbiological diagnosis through a small working channel while protecting the posterior spinal structures and reducing surgical trauma (Wang et al., 2018; George et al., 2024).

Current research on PELD for lumbar spine infections mainly focuses on technical feasibility and short-term symptom relief. However, there is a lack of systematic and in-depth studies on how this minimally invasive procedure affects patients’ perioperative nutritional status and subsequent surgical scope. Some studies evaluate clinical benefits based on outcomes like success rates, adverse events, and recurrence rates (Giordan et al., 2024), but a more comprehensive approach is needed for a local anesthesia minimally invasive surgery. Assessment of spinal infection treatment often relies on isolated time-point indicators such as changes in erythrocyte sedimentation rate (ESR), C-reactive protein (CRP), or visual analog scale (VAS) scores (Liu et al., 2024), which may not fully reflect overall patient recovery, especially the transition from “disease” to “health.” Evaluation of PELD as a potentially non-therapeutic endpoint should integrate multidimensional information from biochemistry (inflammation, nutrition), clinical symptoms (pain, systemic symptoms), and imaging (lesion evolution) for a comprehensive and progressive assessment.

This study retrospectively analyzed clinical data from 27 patients with modified Pola classification (POLA) type IV lumbar spine infections who underwent Stage I PELD-based endoscopic debridement and drainage. The aims were: (1) to preliminarily describe the short-term safety and feasibility of this minimally invasive bridging procedure; (2) to compare changes in inflammation, symptoms, nutrition-related indicators, imaging severity, and anesthesia tolerance during the bridging window before Stage II surgery; and (3) to preliminarily establish an exploratory multidimensional response-assessment framework based on modified POLA classification and clinical process indicators. PELD in this study was not evaluated as an independent curative endpoint or as a substitute for antibiotics, nutritional support, or definitive open surgery. Rather, it was assessed as a planned local debridement, decompression, drainage, and pathogen-sampling procedure within a staged treatment strategy. Because this was a single-arm retrospective self-controlled study, the observed improvements cannot be attributed exclusively to PELD, and potential confounding from antimicrobial therapy, nutritional support, and natural disease evolution should be considered.

## 2 Materials and methods

### 2.1 Study population

A single-center retrospective self-controlled study design was employed. Patients diagnosed with lumbar spine infection and treated with Stage I PELD-based endoscopic debridement and drainage in our department between April 2017 and June 2024 were consecutively included. During this enrollment period, the main indications for Stage I endoscopic bridging intervention, the criteria for subsequent Stage II definitive surgery, and the overall perioperative treatment pathway were kept generally consistent within our department. Nevertheless, given the long study interval, potential time-dependent bias and learning-curve effects were considered in the interpretation of the results.

#### 2.1.1 Inclusion criteria

(1) Definite diagnosis of lumbar spine infection (pyogenic or tuberculous) based on clinical manifestations, elevated inflammatory markers (ESR, CRP), and lumbar spine magnetic resonance imaging (MRI); (2) Poor response to conservative treatment (antibiotics/anti-tubercular therapy) and/or presence of significant epidural or paraspinal abscess; (3) Patients classified as modified POLA Type IV; (4) Patients undergoing PELD in Stage I and lumbar lesion debridement + internal fixation in Stage II; (5) Availability of complete preoperative and postoperative clinical data.

#### 2.1.2 Exclusion criteria

(1) Patients with concomitant malignant tumors, severe cardiac, hepatic, renal, or pulmonary dysfunction, or immunodeficiency disorders (such as acquired immunodeficiency syndrome (AIDS)); (2) Patients with incomplete clinical data or lost to follow-up.

A total of 27 patients met the inclusion criteria for this study. Among them, there were 17 males and 10 females, with an average age of 52.96 ± 16.09 years (range 13–76 years). All 27 patients had lumbar spine infections, with lesions in the following segments: L1/2 (3 cases), L2/3 (5 cases, including 1 case with concurrent L4 vertebral body infection), L3/4 (7 cases, including one case with concurrent L4/5 infection and one case with left paravertebral muscle and bilateral psoas muscle infections), L4/5 (6 cases), L5/S1 (4 cases), T10-L1 (1 case), and T12/L1 (1 case). All patients presented with lower back pain, with 15 cases also experiencing symptoms such as reduced muscle strength or numbness in the lower limbs. X-rays and computed tomography (CT) scans showed narrowed intervertebral spaces, irregular or erosive changes in adjacent endplates, and soft tissue swelling around the vertebrae and epidural area. MRI revealed inflammatory changes in the intervertebral discs, edema in adjacent vertebral bodies, and the formation of paravertebral and epidural abscesses. Contrast-enhanced MRI showed diffuse enhancement in the intervertebral discs and vertebral bodies. Among the 27 patients, 2 had concomitant coronary heart disease (1 of whom also had hypertension, diabetes, and anemia), 4 had hypertension (1 of whom also had uremia and 1 had anemia), 1 had anemia, 2 had type 2 diabetes, 1 had rheumatoid arthritis, 1 had severe aplastic anemia, and 16 had no comorbidities.

### 2.2 Surgical procedure

**Figure 1:**
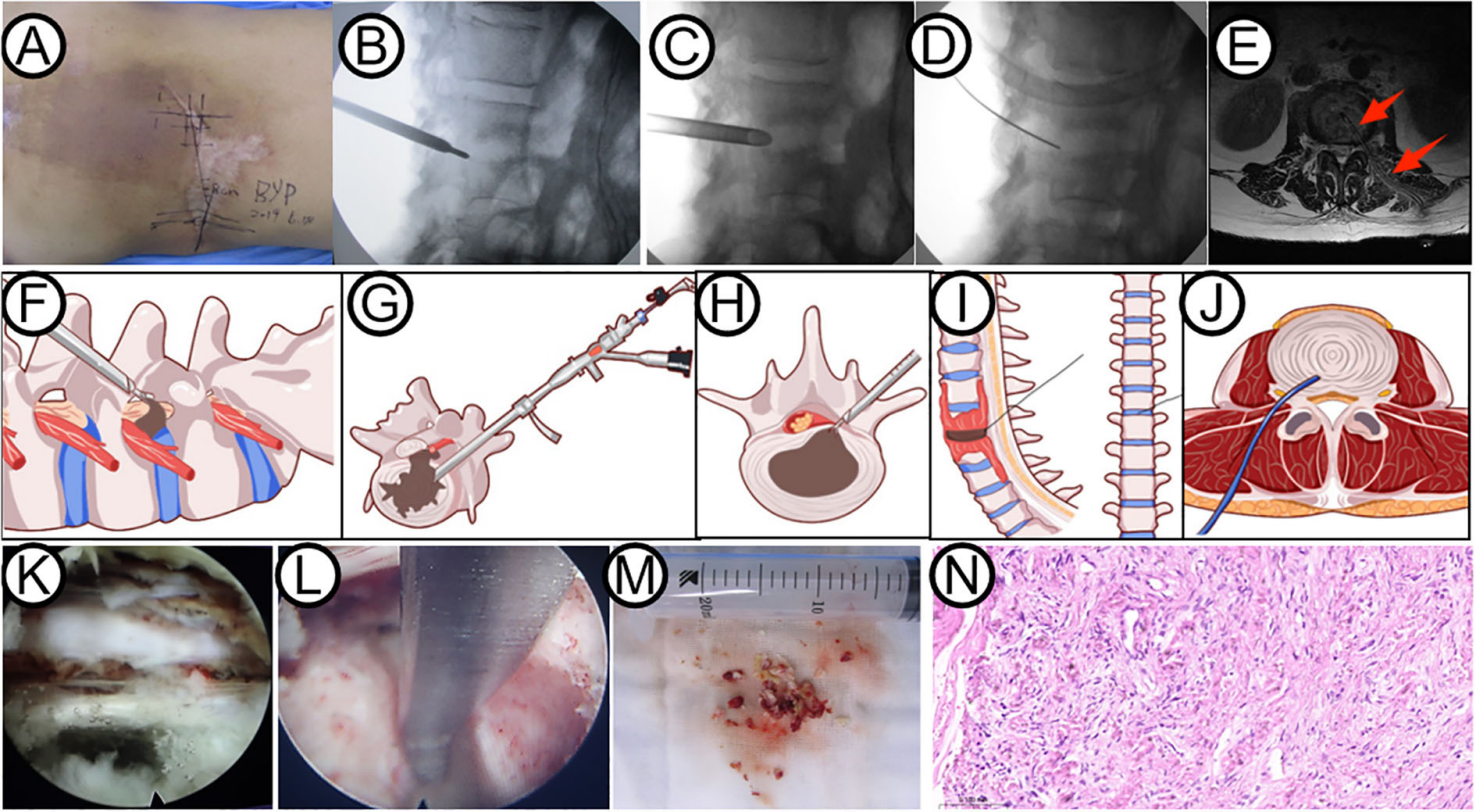
**(A)** represents the needle insertion point and route determined preoperatively under C-arm guidance, while **(B, C)** denote the implanted working channel after puncture and gradual dilation. **(D)** indicates fluoroscopy guidance during replacement for optimal catheter placement. Postoperative MRI **(E)** indicates the drainage tube extending to the intervertebral space. Illustrative diagrams of the surgical process and postoperative drainage tube placement are shown in **(F–J)**. Intraoperative microscopic examination of lesion tissues and clearance status are depicted in **(K, L)**. Pathological tissues obtained during surgery and postoperative hematoxylin and eosin (H&E) staining **(M, N)** confirm inflammatory cell infiltration consistent with lumbar spine infection.

All procedures were performed by the same experienced spine surgery team (**Figure 1**). The German Joymax intervertebral foramen endoscope system was used, along with a disposable radiofrequency plasma knife from Beijing Jiesihui Zhong Company. The procedure was performed under local anesthesia and consisted of percutaneous endoscopic debridement of infected tissue, radiofrequency ablation, extensive irrigation, abscess drainage, tissue sampling, and placement of a negative-pressure drainage tube. Stage I PELD-based endoscopic debridement and drainage was considered when patients had no uncontrolled systemic infection, septic shock, or hemodynamic instability, and when ESR and CRP did not show rapid progressive deterioration. Mild or moderate fever alone was not considered an absolute contraindication if the patient was hemodynamically stable and could tolerate local anesthesia. Throughout the study period, the endoscopic approach, working-channel establishment, debridement principle, irrigation strategy, tissue-sampling protocol, and drainage-tube placement technique remained generally consistent.

### 2.3 Perioperative management

Preoperative: If the patient was clinically stable and could tolerate the bridging procedure, empirical antibiotic initiation before tissue sampling was avoided when feasible to improve pathogen-detection yield. If antibiotics had already been administered before admission or at another hospital, the original regimen was continued initially and subsequently adjusted according to intraoperative microbiological culture and antimicrobial susceptibility results. Nutritional support, including albumin or plasma supplementation, was provided for patients with hypoalbuminemia when clinically indicated. Because preoperative antimicrobial exposure and pathogen type may influence culture positivity and inflammatory-marker trends, these factors were considered potential confounders in the interpretation of treatment response.

Postoperative: For patients with positive microbiological culture results, adequate targeted antimicrobial therapy was continued according to susceptibility testing. For patients with negative culture results, broad-spectrum high-potency antimicrobial therapy was administered until ESR and CRP normalized or stabilized, followed by 4–6 weeks of treatment (Berbari et al., 2015). Early mobilization with lumbar protection was encouraged on the first postoperative day when there was no potential lumbar instability. Nutritional support and appetite/food-intake monitoring were continued. Drainage tubes were generally retained for 5–21 days, and removal criteria included clear drainage fluid, relief of pain symptoms, a significant and stable decrease in ESR and CRP, two consecutive negative cultures from blood and drainage fluid, and drainage volume <10 mL/day for three consecutive days (Fu et al., 2010). After drainage-tube removal, lumbar MRI and CT were performed to assess abscess change and spinal stability, and the modified POLA classification was reassessed to guide the subsequent treatment plan. These perioperative management principles were applied consistently during the study period.

### 2.4 Observation indicators

#### 2.4.1 General indicators

General surgical data included operation time, intraoperative blood loss, intraoperative tissue microbiological culture positivity rate, postoperative VAS and 36-Item Short Form Health Survey (SF-36) scores, and Kirkaldy-Willis criteria. Oswestry Disability Index (ODI) score was used to assess the impact of back pain or leg pain on the patient’s daily activities. This evaluation system assesses pain, daily self-care ability, lifting, walking, sitting, standing, sleeping, sexual activity, social activities, and traveling on a scale of 0 to 5 points for each aspect. Higher scores indicate poorer function, with the total score converted to a percentage: 0-20% indicates mild functional impairment; 21-40% indicates moderate functional impairment; 41-60% indicates severe functional impairment; 61-80% indicates very severe functional impairment; and 81-100% indicates extremely severe functional impairment, requiring bed rest. Postoperative SF-36 score was used to evaluate the patient’s overall health status. Kirkaldy-Willis criteria were used to assess the degree of treatment effectiveness and were divided into excellent, good, fair, and poor.

#### 2.4.2 Procedural indicators

##### 2.4.2.1 Biochemical indicators: foundation of infection control and nutritional reserves

1. Inflammatory Indicators (ESR, CRP): Compare ESR and CRP levels before stage II surgery with those before stage I surgery. CRP is the most sensitive and objective indicator of infection activity. Significance: A rapid decrease in CRP (e.g., >50% per week) is a strong predictor of effective antibiotic therapy. ESR changes slowly, but a sustained increase indicates treatment failure or complications. Literature generally acknowledges CRP’s higher sensitivity and specificity compared to ESR. Multiple studies consider normalization or significant decrease in CRP/ESR as a key criterion for successful non-surgical treatment or safe surgery.
2. Nutritional Indicators (serum albumin, hemoglobin, and body mass index (BMI)): Serum albumin, hemoglobin, and BMI reflect the patient’s nutritional status. Significance: Hypoalbuminemia is an independent risk factor for poor infection prognosis, affecting immune function and tissue healing. Hemoglobin reflects chronic disease anemia and nutritional status. BMI reflects long-term nutritional status. Improving nutritional indicators enhances host resistance and creates conditions for surgery, with the difference in values before and after surgery reflecting the extent of trauma to the patient. Studies confirm a significant correlation between preoperative hypoalbuminemia and post-spinal surgery infection rates, complications, and longer hospital stays.

##### 2.4.2.2 Clinical benefit indicators: direct reflection of patient subjective perception and quality of life

1. Visual Analog Scale (VAS) for Pain: The most important patient demand. Significance: A significant decrease in VAS (usually considered a decrease of ≥2 points or a decrease rate >30%) is direct evidence of treatment effectiveness. It can most intuitively reflect the relief of nerve compression and inflammation reduction. The Visual Analog Scale (VAS, 0–10 points) is used for pain assessment (Verdú-López et al., 2017), where patients rate the pain intensity based on postoperative pain sensation, with 0 indicating no pain and 10 indicating the most severe pain. 1–3 represents mild pain, 4–6 moderate pain, and 7–9 severe pain.
2. Changes in Appetite: A simple functional indicator. Significance: Improvements in appetite are early signs of overall improvement in condition and reduction in symptoms of sepsis, usually coinciding with a decrease in inflammatory markers. Through surveys, appetite changes are categorized as “improved,” “unchanged,” or “worsened.”
3. Body Temperature: Fundamental yet crucial, comparing preoperative highest temperature with temperature changes before phase II surgery. Significance: Normalization of body temperature is a basic indicator of initial control of infection. Prolonged fever should raise concerns about abscess formation, treatment failure, or drug-induced fever.

##### 2.4.2.3 Imaging index (modified POLA classification): an objective basis for determining surgical strategies

**Figure 2:**
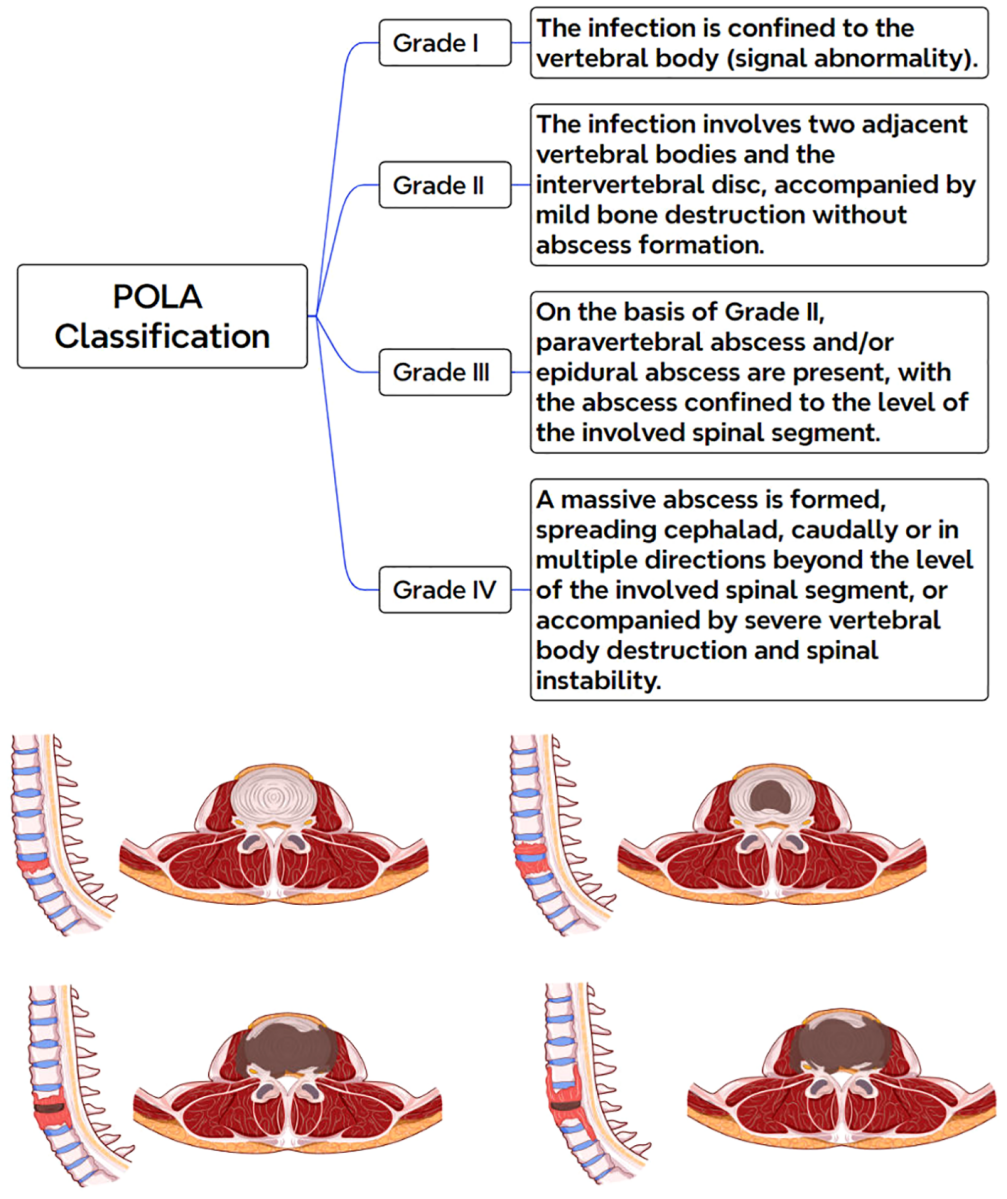
This is the new POLA classification and its corresponding diagram. The classification mainly focuses on the natural cavities involved in the lesion and their extent, thus aiding in the selection of surgical methods and scope.

The modified POLA classification was used as the core imaging index for lesion-extent assessment and staged surgical planning. The original POLA classification provides a framework for grading spinal infection according to anatomical involvement and structural severity. In the present study, the classification was moderately modified to better reflect the characteristics of lumbar spinal infection, including the number of involved anatomical compartments, epidural extension, paravertebral or psoas abscess formation, cranio-caudal spread beyond the index segment, vertebral body destruction, and segmental instability. Therefore, compared with the original classification, the modified version placed greater emphasis on natural-cavity involvement and the practical implication of lesion extent for determining the timing and scope of Stage II surgery. The modified POLA grade was assessed using preoperative MRI and post-PELD MRI/CT. Changes in grade were interpreted as radiological evidence of lesion control or persistence, rather than as a stand-alone marker of definitive cure. The specific grading is shown in **Figure 2**.

Two senior radiologists who were blinded to clinical outcomes independently reviewed preoperative and post-PELD lumbar MRI and CT images and assigned modified POLA grades. In cases of disagreement, consensus was reached through discussion. Two senior spine surgeons further reviewed the imaging findings to confirm the planned scope of Stage II lesion debridement. Formal interobserver reliability statistics, such as weighted kappa or intraclass correlation coefficients, were not calculated in this retrospective cohort, which represents a methodological limitation. Future studies should include multiple independent readers and formally assess both interobserver and intraobserver reliability to verify the reproducibility of the modified POLA classification.

##### 2.4.2.4 Institutional anesthesia-risk assessment (Q Score)

Assessing the patient’s overall condition, tolerance to anesthesia, and suitability for general anesthesia surgery is crucial. A lower anesthesia score indicates reduced anesthesia risks. Some patients may not initially meet the criteria for general anesthesia upon admission, but with treatment, a decreased score signifies reduced risks and the ability to undergo general anesthesia surgery. Prior to surgery, three anesthesiologists should evaluate the patient’s underlying conditions, biochemical indicators, abnormal body temperature, surgical scope, and duration to assess anesthesia risks and calculate an average score. A score of 26–30 indicates high risk and unsuitability for general anesthesia surgery; 20–25 signifies moderate risk, allowing for a comprehensive evaluation based on the patient’s actual condition before proceeding with general anesthesia surgery; and 0–20 indicates low risk, making general anesthesia surgery feasible.

#### 2.4.3 Multiple dimensions of indicators

Individual indicators may present contradictions (e.g., pain relief with persistently high CRP levels); therefore, a comprehensive weighted assessment was explored. The Spinal Infection Bridging Treatment Response Index was designed as a conceptual and exploratory model rather than as a validated prediction tool. The allocation of weights was based on clinical relevance, time sensitivity, and feasibility of data acquisition in relation to the practical goal of successful bridging to definitive treatment. POLA improvement was assigned the highest weight because radiological lesion extent directly influences subsequent surgical planning. CRP and VAS were assigned high weights because they reflect infection activity and patient-centered symptomatic benefit, respectively. Appetite, albumin, hemoglobin, and temperature were incorporated to reflect systemic recovery, nutritional reserve, and infection-control status. These weights were not derived from regression modeling, receiver operating characteristic (ROC) analysis, or an external validation cohort; therefore, the score should be interpreted as a preliminary structured assessment framework and not as an independent clinical decision-making standard.

##### 2.4.3.1 Expert-informed conceptual weight allocation based on literature-supported domains and clinical relevance

**Figure 3:**
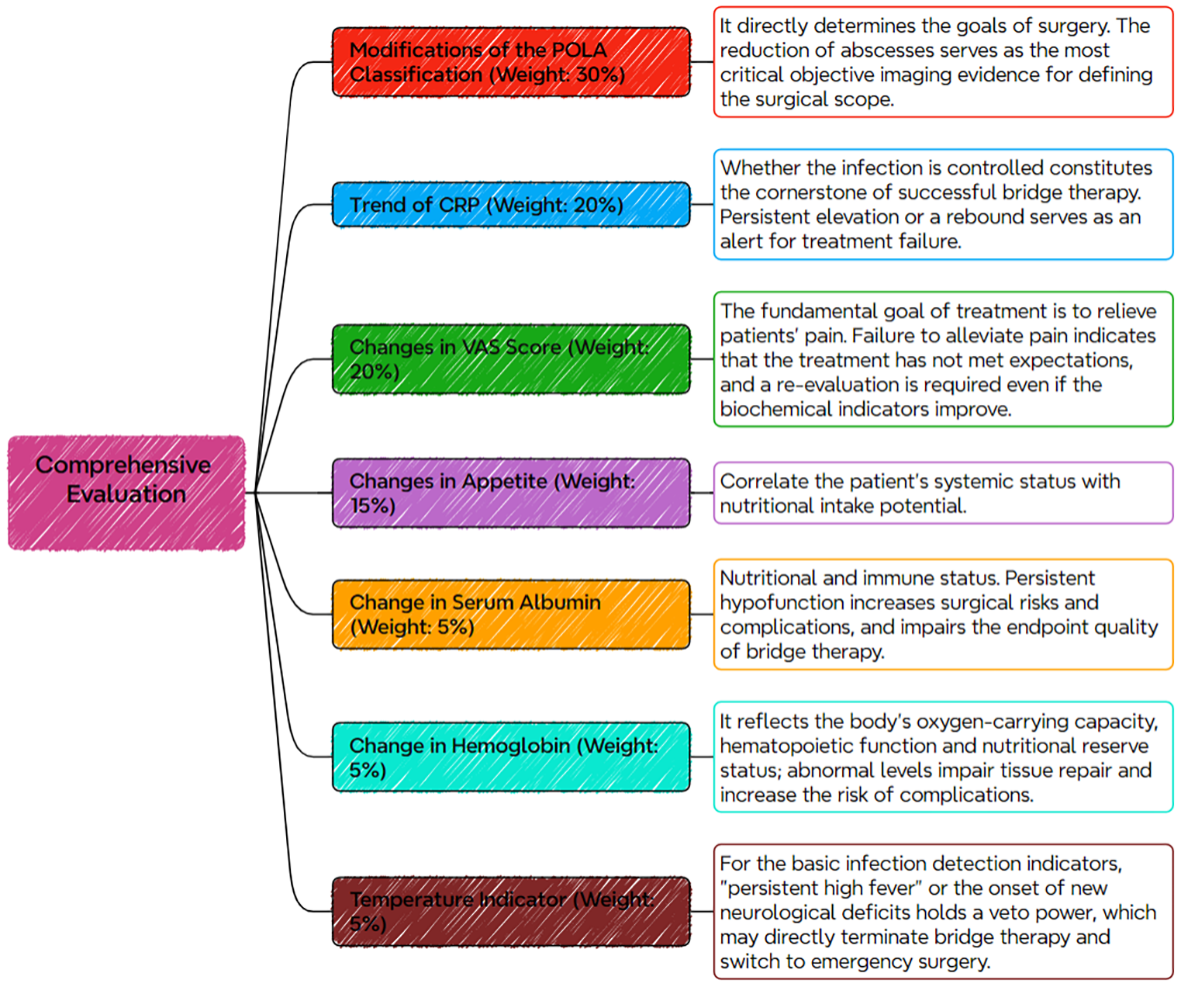
Conceptual structure and investigator-defined weighting of the spinal infection bridging treatment response index. Published evidence supports the clinical relevance of the included domains, whereas the exact numerical weights were not derived from statistical modeling or external validation and should be considered provisional.

The domains included in the Spinal Infection Bridging Treatment Response Index were selected with reference to previously published evidence. The Pola classification was developed as a clinical-radiological framework to support treatment planning in patients with spinal infection (Pola et al., 2017). Current clinical guidelines recommend interpreting changes in erythrocyte sedimentation rate and C-reactive protein together with clinical assessment and, when clinically indicated, follow-up magnetic resonance imaging (Berbari et al., 2015). The visual analog scale is a widely used patient-reported measure for evaluating clinically meaningful changes in pain intensity (Kelly, 2001). Nutritional impairment and hypoalbuminemia have also been associated with adverse outcomes in patients with spondylodiscitis (Gerstmeyer et al., 2025) The conceptual structure and investigator-defined weights are shown in **Figure 3**.

However, the available literature supports the clinical relevance and inclusion of these domains rather than the exact numerical weights assigned to them. The weights used in the present index were investigator-defined on the basis of clinical relevance, time sensitivity, and feasibility of assessment during the bridging window. POLA classification, CRP, and VAS were assigned relatively greater weights because they reflect lesion extent, infection activity, and patient-centered symptomatic response, respectively. Appetite, body temperature, albumin, and hemoglobin were included as exploratory indicators of systemic and nutritional recovery. Therefore, this weighting scheme should be regarded as conceptual and provisional rather than statistically derived or externally validated.

##### 2.4.3.2 Integrated evaluation model (conceptual)

The Spinal Infection Bridging Treatment Response Index (conceptual model) was calculated as follows: Total Score (S) = [(POLA classification improvement, 30 points) + (CRP decrease percentage, 20 points) + (VAS decrease percentage, 20 points) + (serum albumin change, 5 points) + (hemoglobin change, 5 points) + (temperature indicator, 5 points) + (appetite improvement indicator, 15 points)]/10. This score was used only to summarize multidimensional changes during the bridging window and requires further validation before clinical generalization. The detailed scoring criteria for each component of the index are presented in **Table 1**.

**Table 1:** Displays the detailed quantified scoring criteria for each subcategory in the new surgical evaluation system.

POLA classification(30 points)	(1)Decreased by ≥1 grade (30 points) (2)Unchanged (0 points)
CRP(20 points)	(1)Postoperative CRP returned to normal or decreased by ≥50% (20 points) (2)Decreased by <50% (10 points) (3)Increased (0 points)
VAS grade(20 points)	(1)Decreased by >50% (20 points) (2)Decreased by 30–50% (15 points) (3)Decreased by 10–30% (10 points) (4)Decreased by 0–10% (5 points) (5)Unchanged (0 points)
Alb(5 points)	(1)Increased (5 points) (2)Unchanged/Decreased by <10% (3 points) (3)Decreased by >10% (0 points)
Hb(5 points)	(1)Increased (5 points) (2)Unchanged/Decreased by < 10% (3 points) (3)Decreased by > 10% (0 points)
Body temperature(5 points)	(1)≤37.3°C (5 points) (2)>37.3°C (0 points)
Appetite(15 points)	Improvement (15 points)

### 2.5 Statistical analysis

Data analysis was conducted using Statistical Package for the Social Sciences (SPSS), version 21.0. For metric data following a normal distribution, mean ± standard deviation (x ± s) was used, and paired t-tests were applied for pre- and post-operative comparisons. For non-normally distributed data, the median (interquartile range) was used, and the Wilcoxon signed-rank test was employed. Count data were presented as frequencies (percentages) and analyzed using the chi-square test or Fisher’s exact test when applicable. Because the total sample size was limited to 27 patients and pathogen categories were heterogeneous, formal stratified analyses according to pathogen type, tuberculosis status, fungal infection, preoperative antibiotic exposure, or culture-positive versus culture-negative status were not performed. Related findings were therefore interpreted descriptively, and the possibility of residual confounding was acknowledged. A significance level of P<0.05 indicated statistical significance.

## 3 Results

### 3.1 General results

**Figure 4:**
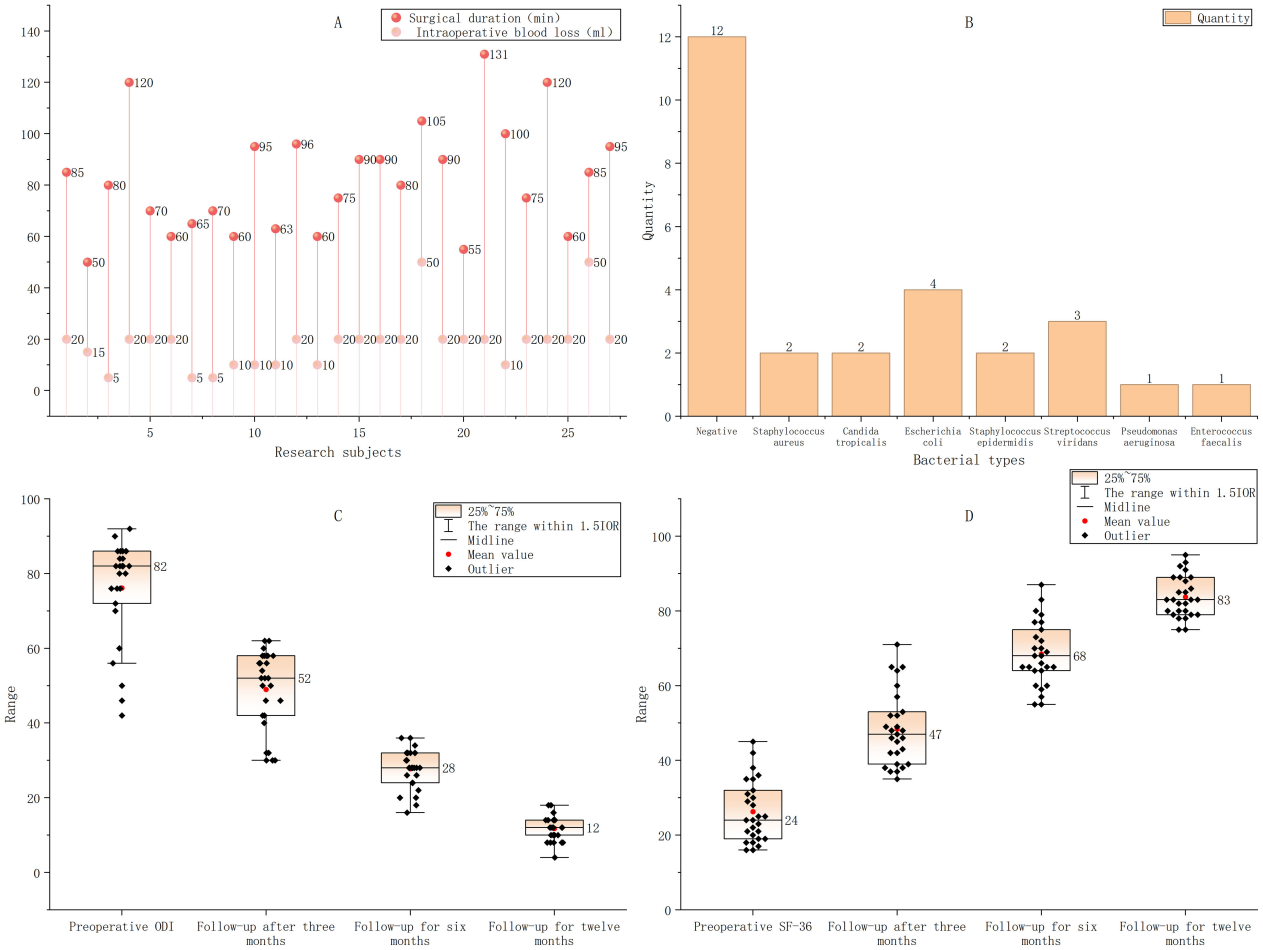
**(A)** displays a scatter plot showing the surgical time and intraoperative blood loss of 27 patients. **(B)**, a bar graph illustrates the bacterial culture results of these patients. **(C)** presents a box plot depicting the ODI scores of the patients before surgery, and at 3, 6, and 12 months postoperatively. Lastly, **(D)** shows a box plot representing the SF-36 scores of the same patients at the corresponding time points.

The general results are summarized in **Figure 4**.

#### 3.1.1 Patient demographics and surgical details

All 27 patients underwent PELD surgery smoothly, with no cases requiring conversion to open surgery. The surgeries proceeded without complications, with an average duration of 82.41 ± 21.14 minutes and an average intraoperative blood loss of 18.52 ± 10.64 ml. There were no occurrences of severe complications such as nerve root or dural sac injuries during the procedures.

#### 3.1.2 Pathogen diagnosis

Tissue samples collected intraoperatively from the 27 patients were submitted for microbiological culture and antimicrobial susceptibility testing, resulting in 15 positive cases and an overall microbiological culture positivity rate of 55.6% (15/27). The identified pathogens included 2 cases of *Staphylococcus aureus*, 2 cases of *Candida tropicalis*, 4 cases of *Escherichia coli*, 2 cases of *Staphylococcus epidermidis*, 3 cases of viridans group streptococci, 1 case of *Pseudomonas aeruginosa*, and 1 case of *Enterococcus faecalis*. Given the small number of cases in each pathogen category, pathogen-specific response comparisons were not statistically reliable and were not performed.

#### 3.1.3 ODI scores, SF-36 scores, and Kirkaldy-Willis criteria were assessed for 27 patients who were followed up at 3, 6, and 12 months post-discharge

Patient recovery post-surgery was evaluated using VAS, ODI scores, SF-36 scores, and Kirkaldy-Willis criteria. The VAS score for back pain at the final follow-up (0.67 ± 0.55) significantly decreased compared to pre-surgery (8.15 ± 1.20). ODI (11.78% ± 3.34%) decreased significantly from pre-surgery levels (76.15% ± 13.54%), while SF-36 [(83.74 ± 5.47) points] increased significantly from pre-surgery [(26.26 ± 8.18) points], all with statistical significance (P < 0.001). Clinical efficacy assessment showed 12 cases (44.4%) as excellent and 15 cases (55.6%) as good, with a total excellent/good rate of 100%.

### 3.2 Procedural outcomes

#### 3.2.1 Biochemical indicators

**Figure 5:**
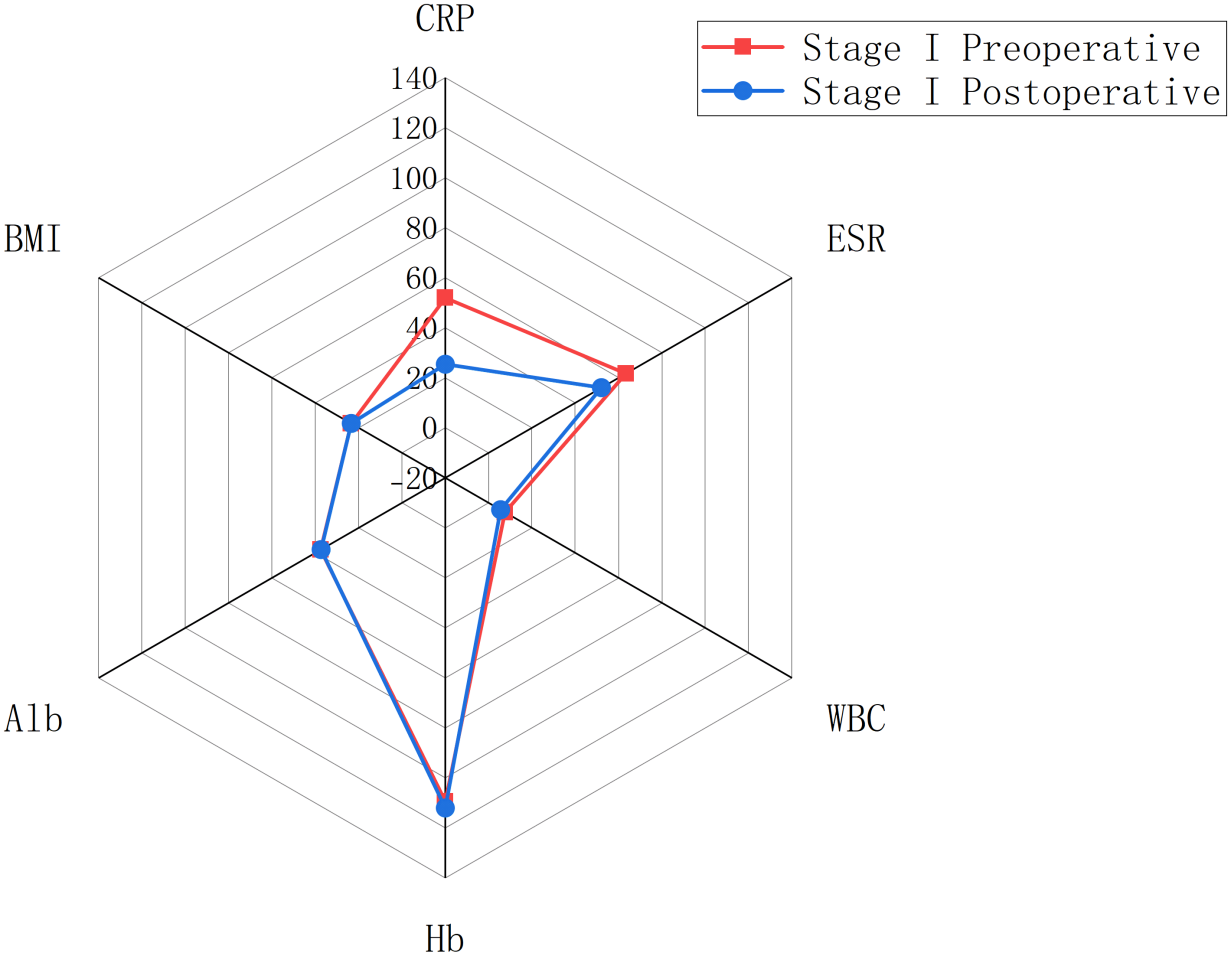
The radar chart in figure illustrates the differences in CRP, ESR, and white blood cell count (WBC), before and after surgery for 27 patients, indicating no significant variances in BMI, albumin (Alb), and hemoglobin (Hb), which are nutritional indicators.

Changes in the biochemical indicators are shown in **Figure 5**.

##### 3.2.1.1 Inflammatory markers

Postoperative CRP [(25.45 ± 23.19) mg/L] significantly decreased compared to preoperative CRP [(51.95 ± 36.88) mg/L], with a statistically significant difference (P<0.001); postoperative ESR [(52.07 ± 27.14) mm/1 h] showed a significant decrease compared to preoperative ESR [(63.33 ± 33.37) mm/1 h], with a statistically significant difference (P = 0.015<0.05).

##### 3.2.1.2 Nutritional Indicators

There were no significant changes in postoperative serum albumin [(37.34 ± 13.32) g/L] compared to preoperative levels [(37.57 ± 12.47) g/L], with no statistical significance (P = 0.786>0.05); postoperative hemoglobin [(112.00 ± 29.01) g/L] did not significantly differ from preoperative levels [(109.37 ± 30.02) g/L], with no statistical significance (P = 0.392>0.05); postoperative BMI [(23.54 ± 3.77) kg/m^2^] did not significantly change compared to preoperative levels [(23.39 ± 3.71) kg/m^2^], with no statistical significance (P = 0.549>0.05). Therefore, it can be observed that PELD surgery did not have a significant impact on the nutritional status of patients.

#### 3.2.2 Clinical outcome indicators

**Figure 6:**
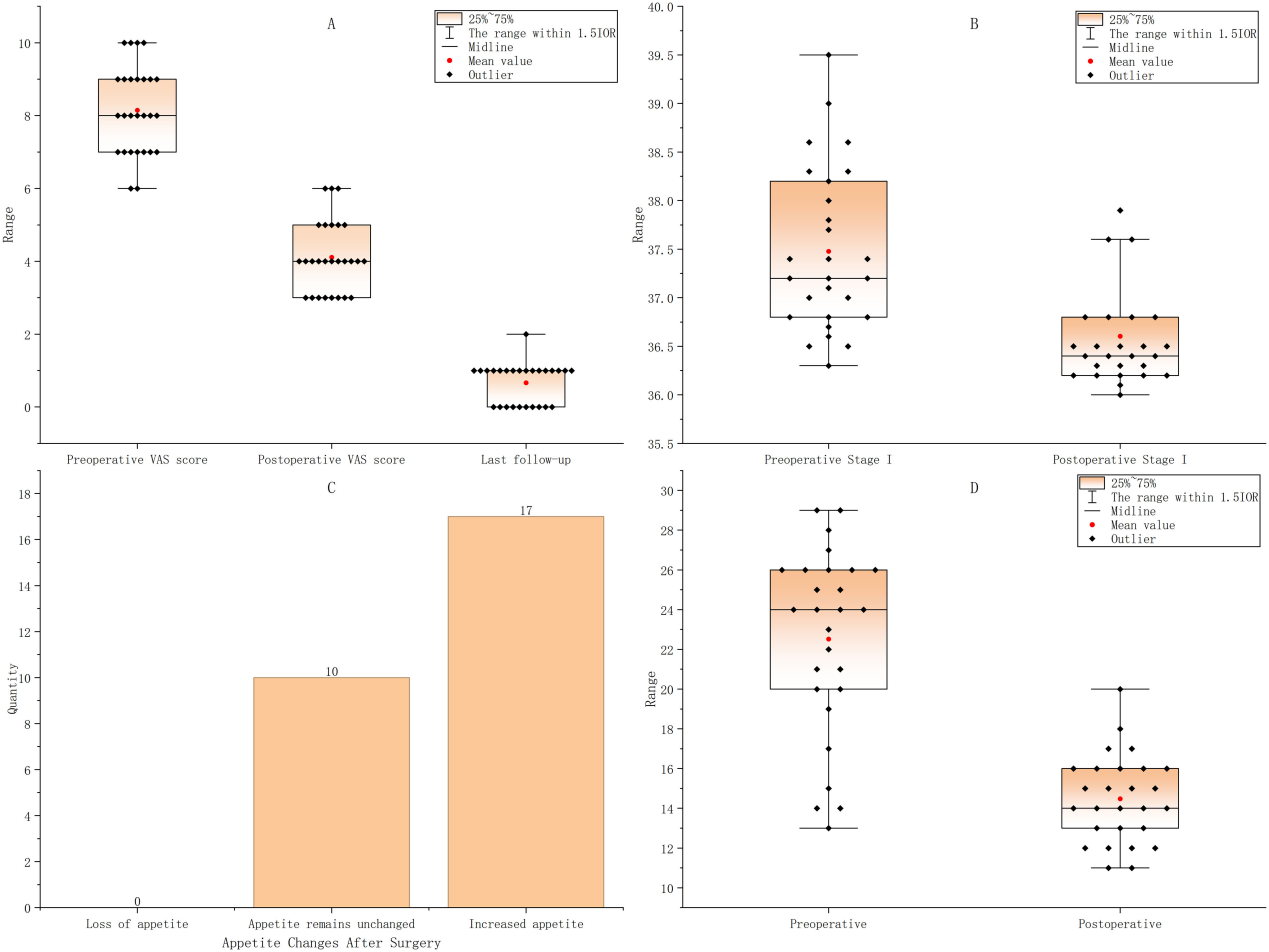
**(A)** is a box plot of the visual analogue scale (VAS) scores of 27 patients before surgery, after surgery and at the last follow-up; **(B)** is a box plot showing the body temperature of these 27 patients before and after the primary surgery; **(C)** is a bar chart illustrating the changes in appetite of the 27 patients after surgery; **(D)** is a box plot reflecting the anesthesiologists’ scoring results for the same 27 patients before and after surgery.

The clinical outcome indicators are summarized in **Figure 6**.

##### 3.2.2.1 Pain visual analog scale

Postoperatively, the VAS score at 3 days after surgery [(4.11 ± 0.97) points] significantly decreased compared to preoperative [(8.15 ± 1.20) points].

##### 3.2.2.2 Temperature changes

Before the surgery, 13 patients had a fever (temperature >37.3 °C). The average preoperative temperature was [37.48 ± 0.83]°C, which decreased to [36.60 ± 0.53]°C postoperatively, showing a statistically significant difference (P<0.001).

##### 3.2.2.3 Appetite

After the surgery, a survey on appetite revealed that 17 out of 27 patients (62.96%) reported a significant increase in appetite compared to before the operation, while 10 patients (37.04%) stated no change, with none experiencing a decrease in appetite.

##### 3.2.2.4 Anesthesia score (Q)

The postoperative anesthesia Q score [(14.48 ± 2.19) points] decreased compared with the preoperative score [(22.52 ± 4.68) points], with a statistically significant difference (P<0.001). This finding suggests that the overall anesthesia-risk profile improved during the bridging window after Stage I intervention. However, the Q score was an institutional anesthesia-risk assessment tool rather than an externally validated universal scoring system, and its results should be interpreted in that context. .

#### 3.2.3 Improved POLA classification-based efficacy evaluation

**Figure 7:**
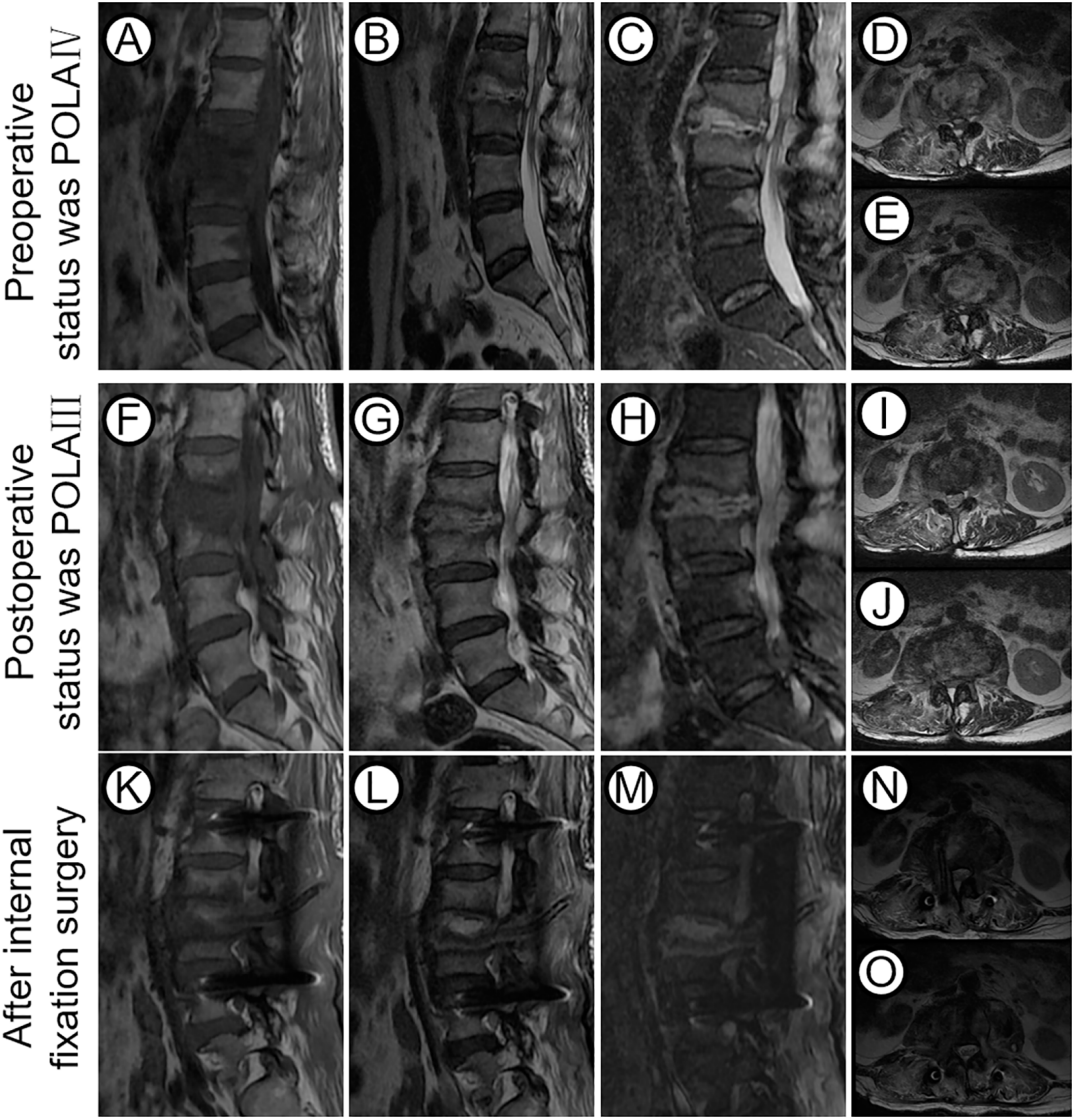
Illustrates the new POLA classification corresponding to a 50-year-old female patient with lumbar spine infection before **(A–E)**, after PELD surgery **(F–J)**, and after internal fixation surgery **(K–O)**. Preoperative lumbar spine MRI images **(A–C)** and cross-sections **(D, E)** show bone destruction and abscess formation in the vertebral bodies and intervertebral disc at L2-L3, with an epidural abscess causing nerve compression, classified as POLA type IV. Postoperative lumbar spine MRI images **(F–H)** and cross-sections **(I, J)** after endoscopic irrigation and drainage show a significant reduction in the abscess at L2-L3, with resolution of the epidural abscess, classified as POLA type III. Follow-up lumbar spine MRI images **(K–M)** and cross-sections **(N, O)** after stage II debridement and internal fixation surgery demonstrate complete resolution of the abscesses, leading to a downgrade of the POLA classification to type II.

Preoperative MRI assessments revealed that all patients had a modified POLA grade of IV. Following the initial PELD-based endoscopic debridement and drainage phase, a subsequent MRI was conducted before the second-stage surgery, showing a decrease of at least one grade in all patients (**Figure 2**). Specifically, 17 cases decreased to grade III, and 10 cases decreased to grade II. The difference in POLA grade distribution between preoperative and post-PELD stages was statistically significant (Z=-4.710, P<0.001). These findings indicate radiological downgrading of lesion extent after bridging intervention. However, because no parallel control group was included, this improvement should be interpreted as being associated with the integrated staged treatment process rather than as definitive proof of an isolated PELD effect. Representative imaging changes are shown in **Figure 7**.

#### 3.2.4 Multidimensional evaluation

**Figure 8:**
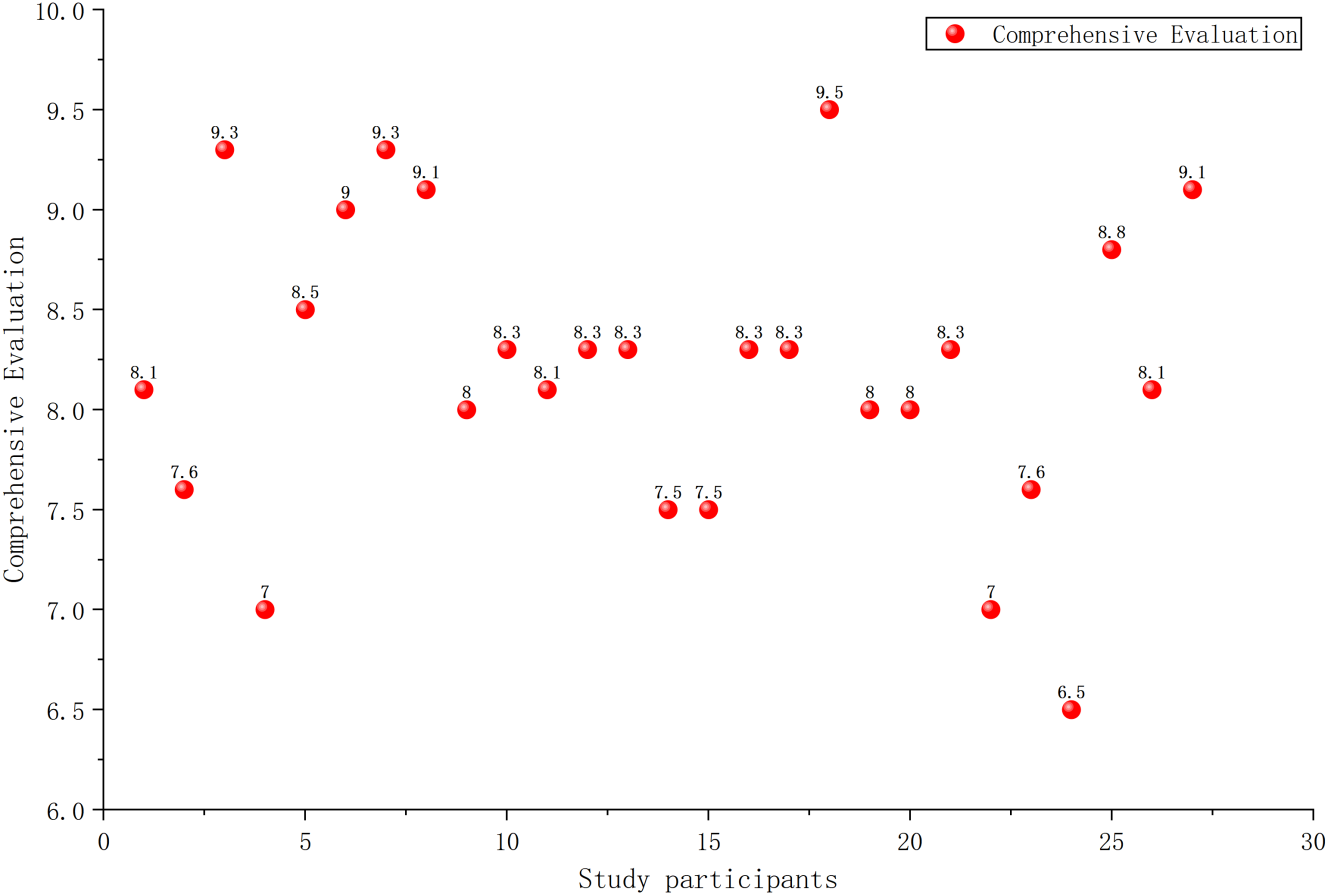
Shows a scatter plot of the comprehensive scores (S) of 27 patients, with the highest total score reaching 9.5 points and the lowest being 6.5 points. The average postoperative score is [8.20 ± 0.75] points.

Interpretation: Following Phase I endoscopic surgery, relevant indicators of 27 patients with spinal infections were analyzed. The total score S was calculated using a comprehensive evaluation model. Postoperatively, the highest total score S among patients reached 9.5 points, while the lowest was 6.5 points. The average value of S post-surgery was [8.20 ± 0.75]. (**Figure 8**).

In this exploratory framework, S > 6 points was considered suggestive of a favorable bridging response and may support preparation for elective Stage II surgery or continued conservative treatment when clinically appropriate. A score between 4 and 6 points suggested an intermediate response requiring reassessment of weak dimensions, such as insufficient infection control or inadequate pain relief. A score <4 points suggested poor bridging response and should prompt reconsideration of antimicrobial strategy, evaluation for resistant or hidden infection foci, or earlier definitive surgical intervention. These cut-off values were preliminary and require external validation.

## 4 Discussion

Through systematic analysis of clinical data from 27 patients with lumbar spine infections before and after Stage I PELD-based endoscopic debridement and drainage, this study preliminarily suggested that this minimally invasive procedure may contribute to early clinical stabilization, including infection control and symptom relief, when used as part of a staged integrated treatment pathway (Verdú-López et al., 2017). The study also explored perioperative nutrition-related indicators and proposed a multidimensional clinical response-assessment framework. Given the retrospective self-controlled design, these findings should be interpreted cautiously and should not be considered definitive evidence that PELD alone caused the observed improvements.

### 4.1 Minimally invasive value of PELD in the treatment of lumbar vertebral infections

Lumbar vertebral infections are considered deep tissue infections with limited antibiotic effectiveness due to poor blood supply in the intervertebral space. As the condition progresses, it can lead to structural damage of vertebral bodies and intervertebral discs, potentially causing abscesses and nerve compression. If the pathogens enter the bloodstream, it can lead to systemic complications such as sepsis, pyemia, and even septic shock. The treatment goals for lumbar vertebral infections are: 1. Alleviate pain; 2. Maintain or restore spinal stability; 3. Prevent or reverse neurological deficits (Yang et al., 2014). The clinical surgical strategies for infectious spinal diseases mainly focus on controlling the infection, altering the local microenvironment of the lesion, and maintaining spinal stability. With advancements in minimally invasive spinal surgery techniques (Moschos et al., 2025), PELD has gradually been applied in the clinical treatment of lumbar infectious diseases due to its advantages of shorter operation time, less bleeding, and faster recovery (Chen et al., 2021; Huang et al., 2022; Rajkumar and Li, 2024). PELD lesion clearance combined with irrigation and drainage can provide microbiological and pathological specimens, guiding the rational use of antibiotics and effectively changing the microenvironment of the infected lesion to rapidly control the infection, relieve pain, and fever symptoms (Ito et al., 2007).

This study suggested the minimally invasive characteristics of the Stage I endoscopic procedure, with an average operation time of 82.41 ± 21.14 min and mean blood loss of 18.52 ± 10.64 mL. These findings are consistent with the clinical rationale that local endoscopic debridement and drainage may reduce surgical stress compared with more extensive open procedures (Duarte and Vaccaro, 2013). Nevertheless, because the study lacked a contemporaneous open-surgery control group, direct comparative claims regarding superiority over traditional open surgery should be avoided. The observed decreases in ESR and CRP and the reduction in pain may reflect the combined effects of endoscopic drainage, antimicrobial therapy, nutritional support, and natural disease evolution during the staged treatment process.

### 4.2 The positive impact of PELD on patients’ nutritional risk

Malnutrition is an independent risk factor for the prognosis of infectious diseases. The results of this study indicate that there were no statistically significant differences in albumin, hemoglobin, and BMI of postoperative patients compared to preoperative values, suggesting a minimal impact of PELD surgery on patients’ nutritional status. Reasons for the lack of statistical significance in the analysis of differences may include:

Early observation time point: Nutritional recovery is a relatively slow process. This study assessed patients at 2 weeks postoperatively, a time when the body may still be in the early stages of synthetic metabolism. Longer follow-ups (e.g., 1, 3 months postoperatively) may capture more statistically significant improvements.

Indirect promotion effect: The improvement in nutritional status by PELD may be primarily indirect. By promptly controlling the source of infection (fever reduction) and relieving pain, there is a significant improvement in the overall physiological status and appetite of patients, creating optimal conditions for self-nutrient intake. The general increase in appetite and normalization of body temperature observed in this study are important prerequisites and strong driving forces for improving the nutritional status of some patients.

Sample size limitation: The sample size of this study (n=27) may be insufficient to detect relatively subtle changes in nutritional indicators or to support robust stratified analyses by pathogen type, preoperative antimicrobial exposure, or disease subtype. Therefore, PELD-based endoscopic debridement and drainage should be interpreted as potentially helping to preserve nutritional reserve and create favorable conditions for subsequent recovery, rather than as directly reversing malnutrition.

In this group, all 27 cases of lumbar spine infection underwent Stage I PELD-based endoscopic debridement and drainage, followed by radiological downgrading of modified POLA classification before Stage II surgery. This finding suggests that the bridging procedure was associated with reduced lesion extent on imaging and may have facilitated subsequent surgical planning. However, in the absence of a direct control group, the decrease in POLA grade cannot be attributed exclusively to PELD. The procedure carries lower immediate surgical burden than extensive open surgery, and no severe intraoperative complications such as nerve root injury, meningitis, or worsening lower-limb neurological symptoms were observed in this cohort. The clinical significance of modified POLA downgrading should be verified in future controlled studies.

The modified POLA system provides a structured radiological description of lesion evolution. In this study, the decrease in POLA grading was interpreted as supportive evidence of abscess reduction and lesion control during the bridging window. Biomarkers such as ESR/CRP and body temperature reflected biochemical and physiological responses to infection management, whereas clinical symptoms such as VAS and appetite reflected patient-centered recovery. Nutritional indicators such as albumin, hemoglobin, and BMI were used to describe metabolic reserve but did not significantly improve in the short-term observation window. The advantage of this system lies in its comprehensiveness and dynamic nature, but it remains an exploratory framework requiring external validation.

### 4.3 Methodological limitations and bias control

First, this was a single-center retrospective self-controlled study with only 27 patients and no parallel open-surgery or conservative-treatment control group. Therefore, the observed improvements cannot be ascribed exclusively to PELD-based endoscopic debridement and drainage. Antimicrobial therapy, nutritional support, drainage management, and spontaneous disease evolution may all have contributed to clinical improvement.

Second, pathogen heterogeneity and preoperative anti-infective exposure may have influenced inflammatory markers, culture positivity, and treatment-response assessment. Because of the limited sample size and small numbers within individual pathogen categories, formal stratified analyses were not performed. Future larger studies should stratify patients by pyogenic, tuberculous, fungal, culture-positive/culture-negative status, antibiotic-exposure history, and disease duration.

Third, the modified POLA classification and the Spinal Infection Bridging Treatment Response Index were exploratory tools. Interobserver reliability of modified POLA grading was not formally assessed, and the weights of the response index were derived from clinical logic rather than statistical modeling or external validation. Future studies should test reproducibility, predictive validity, optimal weighting, and clinically meaningful cut-off values in independent cohorts.

Fourth, the enrollment period extended from April 2017 to June 2024. Although all procedures were performed by the same experienced spine surgery team and the major surgical indications, endoscopic approach, tissue-sampling protocol, irrigation-drainage strategy, and antimicrobial-management principles remained generally consistent, time-dependent bias and learning-curve effects cannot be completely excluded. Because of the limited sample size, chronological subgroup analysis was not performed; future studies should compare early and later cases to evaluate the influence of technical maturation.

Fifth, Although published evidence supports the clinical relevance of the selected imaging, inflammatory, symptomatic, and nutritional domains, the numerical weights and cut-off values of the index were not derived from regression modeling, Delphi consensus, receiver operating characteristic analysis, or external cohort validation. Further studies are required to optimize and validate the weighting structure before the index can be used for clinical decision-making.

## 5 Conclusion

PELD-based percutaneous endoscopic debridement, irrigation, and drainage may serve as a feasible minimally invasive bridging procedure for selected patients with modified POLA type IV lumbar spinal infection who require staged treatment. In this cohort, the procedure was associated with early pain relief, decreased inflammatory markers, improved body temperature, radiological downgrading of lesion extent, and improved anesthesia tolerance before Stage II surgery. However, these findings should be interpreted as preliminary evidence from a retrospective self-controlled cohort rather than definitive proof of an independent curative effect. The proposed multidimensional evaluation system may provide a structured framework for describing early bridging response, but its reproducibility, weighting rationale, and predictive validity require further validation in prospective controlled studies.

Future research should include larger prospective multicenter cohorts, standardized antimicrobial and nutritional protocols, formal reliability testing for modified POLA classification, external validation of the response index, and appropriately controlled comparisons with direct open surgery or conservative treatment to more accurately determine the independent contribution of this bridging strategy.

## Data Availability

The original contributions presented in the study are included in the article/supplementary material. Further inquiries can be directed to the corresponding author/s.
